# Vessel trajectory classification via transfer learning with Deep Convolutional Neural Networks

**DOI:** 10.1371/journal.pone.0308934

**Published:** 2024-08-26

**Authors:** Hwan Kim, Mingyu Choi, Sekil Park, Sungsu Lim

**Affiliations:** 1 Department of Computer Science and Engineering, Chungnam National University, Daejeon, Korea; 2 Korea Research Institude of Ships & Ocean Engineering (KRISO), Daejeon, Korea; University of Manitoba, CANADA

## Abstract

The classification of vessel trajectories using Automatic Identification System (AIS) data is crucial for ensuring maritime safety and the efficient navigation of ships. The advent of deep learning has brought about more effective classification methods, utilizing Convolutional Neural Networks (CNN). However, existing CNN-based approaches primarily focus on either sailing or loitering movement patterns and struggle to capture valuable features and subtle differences between these patterns from input images. In response to these limitations, we firstly introduce a novel framework, Dense121-VMC, based on Deep Convolutional Neural Networks (DCNN) with transfer learning for simultaneous extraction and classification of both sailing and loitering trajectories. Our approach efficiently performs in extracting significant features from input images and in identifying subtle differences in each vessel’s trajectory. Additionally, transfer learning effectively reduces data requirements and addresses the issue of overfitting. Through extended experiments, we demonstrate the novelty of proposed Dense121-VMC framework, achieving notable contributions for vessel trajectory classification.

## Introduction

The Automatic Identification System (AIS) is a self-reporting mechanism for vessels, designed to enhance maritime safety by sharing information about a ship’s movements to assess its strategic intentions [1]. A ship is automatically able to transmit movement and status information such as speed, location, and navigation [2]. This AIS system offers an efficient way to identify and analyze vessel activities [3]. Typically, AIS data is presented as a sequence of points representing ship’s trajectory. Even though AIS data is advantageous for computer storage and management of extensive AIS records, it still remains challenging to analyze a ship’s behavior [4]. Owing to the widespread usage of AIS devices on board, AIS data for analyzing ship’s movements has become easily accessible with large sample sizes. Nevertheless, a majority of AIS data primarily reflects the standard navigation progress of ships, consistently maintaining speed and course on navigation.

Regarding this matter, [5] have introduced a conceptual perspective on vessel trajectories known as the “Stop/Move” model. This framework characterizes ship movements as a series of stopping and moving. In the stopping phase, a ship remains stationary or moves steadily in a specific area. On the other hand, the moving phrase represents a ship’s movement between two stopping points. In addition, [6] categorizes the moving phase into two sub-types: sailing straight and turning with regard to course change.

**Fig 1:**
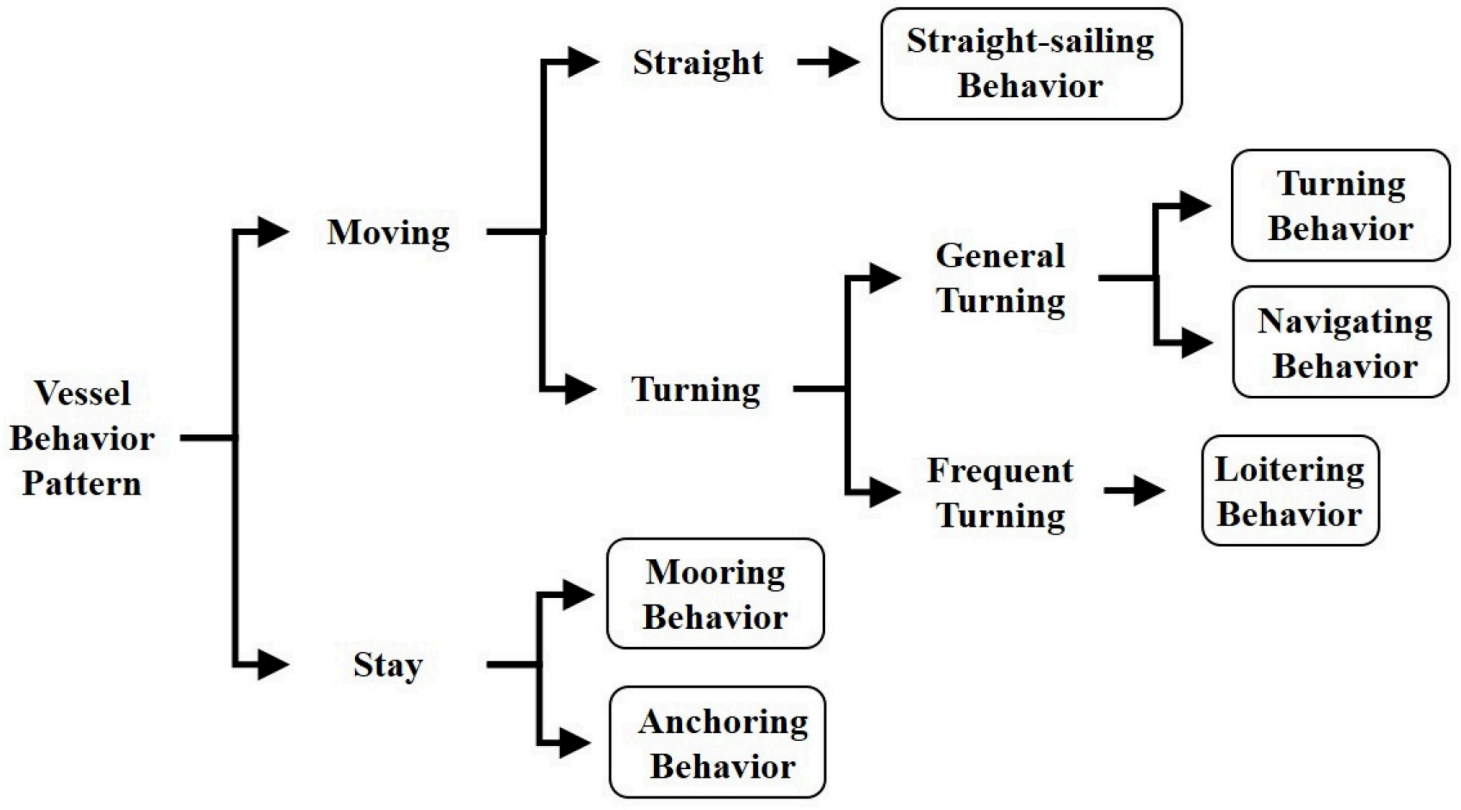
Taxonomy of basic ship behavior patterns.

The ship’s navigating state represents that the vessel must either adjust its speed or alter its course to prevent collisions and only a small portion of an entire AIS data is the sailing state [7]. Intuitively, vessel collisions may happen when a ship turns its directions unexpectedly or frequently. To delve into the study of preventing ship’s collision, the first stage is to categorize the turning behaviors into three categories: straight-sailing, general turning, and navigating. [8] discover that a wide range of vessels frequently engage in repetitive moving patterns on its courses. These particular ship movements are very similar to the way that humans might wander or go around in a specific place, and it’s referred as loitering behavior. When it comes to analysis of ship’s moving behaviors, there have been limited efforts for examining the distinct characteristics of moving patterns as well as loitering behaviors in an area. The act of a ship loitering behaviors in a specific area may signify its significant interests in that place or something related within this place [8]. As a result, classifying vessel trajectories based on both sailing and loitering patterns can improve maritime safety and facilitate efficient navigation and traffic management in congested waterways or ports [9]. Fig 1 provides a clear depiction of these ship movement patterns.

Vessel trajectory classification is one of the most fundamental research area using AIS data as the outcomes of this classification hold significant importance in various domains. These include ocean surveillance and conservation [10], vessel management enhancement [11], and navigation safety protection [12]. Furthermore, the classification results can fortify fishery management and promote conservation of the ocean ecosystem by identifying and monitoring illegal, unreported, and unregulated (IUU) fishing boats and actions associated with illegal over-fishing [13–15]. In addition, these classification outcomes also have the potential to aid navigators and coastal authorities in identifying suspicious or unsafe vessel activities and potential threats. This action can be achieved by comparing the categorized patterns of ships with those identifying results reported by the vessels [16, 17].

In recent years, many researchers have attempted to solve this ship trajectory classification with statistical approaches. [18] introduce a trajectory classification approach known as TraClass. This method produces a set of features in a hierarchical structure through a combination of area- and route-based clustering. The area-based clustering focuses on capturing broader area characteristics without movement patterns, while the trajectory-based clustering concentrates on fine-grained features by considering ship movement patterns. The combination of these two clustering methods facilitates the straightforward identification of moving objects. [19] firstly distinguish both ship movement and static state by employing linear segmentation. Subsequently, it establishes ship movement trajectory similarities using these segments as kernels, employing the Support Vector Machine (SVM) for classifying ship movements. This method is most effective within a specific maritime area, and the accuracy of the kernel function depends heavily on the availability of huge sample data to achieve high precision. [6] employ a logistic regression model to create a ship classifier, utilizing features directly derived from ship AIS trajectories. This method firstly introduces a classifier to categorize ship movements into three types and subsequently develops three distinct logistic regression models for trajectory classification. This approach proves its effectiveness in discerning various movements, particularly on large datasets. [20] present a Least-squares Cubic Spline Curves Approximation (LCSCA) approach to reconstruct AIS points by using characteristics of AIS datasets. Lp-norm sparse representation technique is proposed to identify the ship movement behaviors.

With the great successes of deep learning in many domains [21], some researchers [14, 16, 22, 23] have adopted deep learning techniques for vessel trajectory classification, and accomplished novel performance. For example, [24] introduces a CNN-based approach for vessel sailing movement classification. Specifically, this approach detects three types of movement patterns from AIS data and generates trajectory images of each pattern. Then, multi-CNN layers are used to extract features from these images and predict movement classes. Similarly, [8] proposes a CNN-based framework for detecting and classifying four different loitering movements of vessels on images generated from AIS data. Likewise, these AIS images are identified by a stacked CNN model.

However, these previous CNN-based methods have two drawbacks on vessel movement classification. First, either sailing movements or loitering patterns are considered for trajectory classification. In order to complete the aforementioned maritime safety and efficient navigation and traffic management, identification of whole moving patterns will be needed intuitively. Hence, we attempt to classify the entire movement patterns of vessels with well-designed convolutional networks. Second, the previous methods are not capable of effectively distinguishing subtle differences in trajectories since the model is not deep enough to extract valuable features from AIS images. In order to make deep convolutional networks, these methods simply stack the multiple convolutional layers. However, it brings about an over-smoothing problem that the performance of model is not improved as the number of layers increases and overfitting issue due to the small amount of training images.

To address these issues, we propose Deep Convolutional Neural Networks (DCNN) based framework, Dense121-VMC, for efficient vessel trajectory classification with transfer learning, which is the reuse of a pretrained model on a new task. First of all, the proposed Dense121-VMC is capable of extracting more valuable features from input images. Based on these important features, subtle differences of each vessel trajectory become more distinguishable and our model can efficiently identify the ship movement patterns. Moreover, transfer learning technique reduces data requirements and it could solve the overfitting issue. The architectures of pretrained models are carefully composed and optimized, often achieving state-of-the-art performance. Hence, utilizing these models as a backbone network allows us to benefit from cutting-edge capabilities without starting from the baseline.

In summary, contributions of our paper are as follows:

- To the best of our knowledge, our paper is the first work for identifying both sailing and loitering trajectories simultaneously with transfer learning technique.
- We propose a well-designed DCNN-based framework, Dense121-VMC, to detect moving patterns on trajectory and to extract fine-grained features to distinguish the subtle differences of vessel movement patterns.
- To prove the effectiveness of our method, we conduct comprehensive experiments on different CNN-based methods and models.

## Related work

In this section, we sum up previous works for vessel movement classification and image classification methods based on machine learning or deep learning methods.

### Vessel trajectory classification

Vessel trajectory classification is to categorize trajectories into different types using AIS spatio-temporal data. Researchers have devoted significant attention to exploring this issue through the machine learning or deep learning techniques. In terms of machine learning approaches, various method has been employed to improve the classification tasks. For instance, [25] introduce an algorithm utilizing conditional random fields to identify fishing activities on historical AIS data. [6] adopt the logistic regression algorithm to construct a classifier using trajectory features for distinguishing between fishing and cargo ship trajectories. [26] first employ interacting multiple models to filter AIS data, classifying ship moving courses by using a multi-class binary decision tree. [27] propose a classifier based on random forest to identify types of vessels, such as cargo, tanker, and fishing boats. Encouraged by the notable achievement of deep learning, researchers have devised various methods based on deep learning techniques for classifying vessel trajectories. [28] present a framework using autoencoders to detect fishing activities from vessel trajectories. [16] utilize CNN layers to discern features of vessels and identify type of ships, such as passenger, fishing, tanker, and cargo types, drawing on AIS kinematic data. [22] introduce a deep learning framework using CNN layers for vessel monitoring for anomaly detection. [8, 24] introduce an algorithm for detecting vessel moving trajectories and identifying ship movement patterns, based on a convolutional neural network. [14] present a straightforward and efficient CNN-based model for trajectory classification. This model is trained using a set of invariant spatio-temporal information exploited from the moving characteristics of vessel. [23] convert vessel trajectory characteristics into images and leverage deep learning techniques to categorize vessel activities. These deep learning methods successfully demonstrate its superior performance to traditional machine learning techniques.

### Image classification with CNN

Convolutional Neural Networks (CNN) holds significant importance in the deep learning area. In the era of big data, CNN distinguishes itself from traditional approaches by effectively leveraging vast amounts of data to yield promising outcomes. Image classification is to identify an image to a specific class category, and CNN shows outstanding performance in this tasks. CNN offers several advantages. Firstly, it employs local connections, where each neuron is linked to only a small subset of neurons in the previous layer. This process effectively reduces the number of parameters and speeds up its convergence. Secondly, it utilizes weight sharing, enabling a group of connections to share the same weights, further reducing the number of parameters. Lastly, it leverages down-sampling through a pooling layer, which can reduce the amount of data, containing valuable information. These three advantages above establish CNN as one of the most fundamental algorithms in the field of deep learning and image classification task [21, 29].

## Methodology

**Fig 2:**
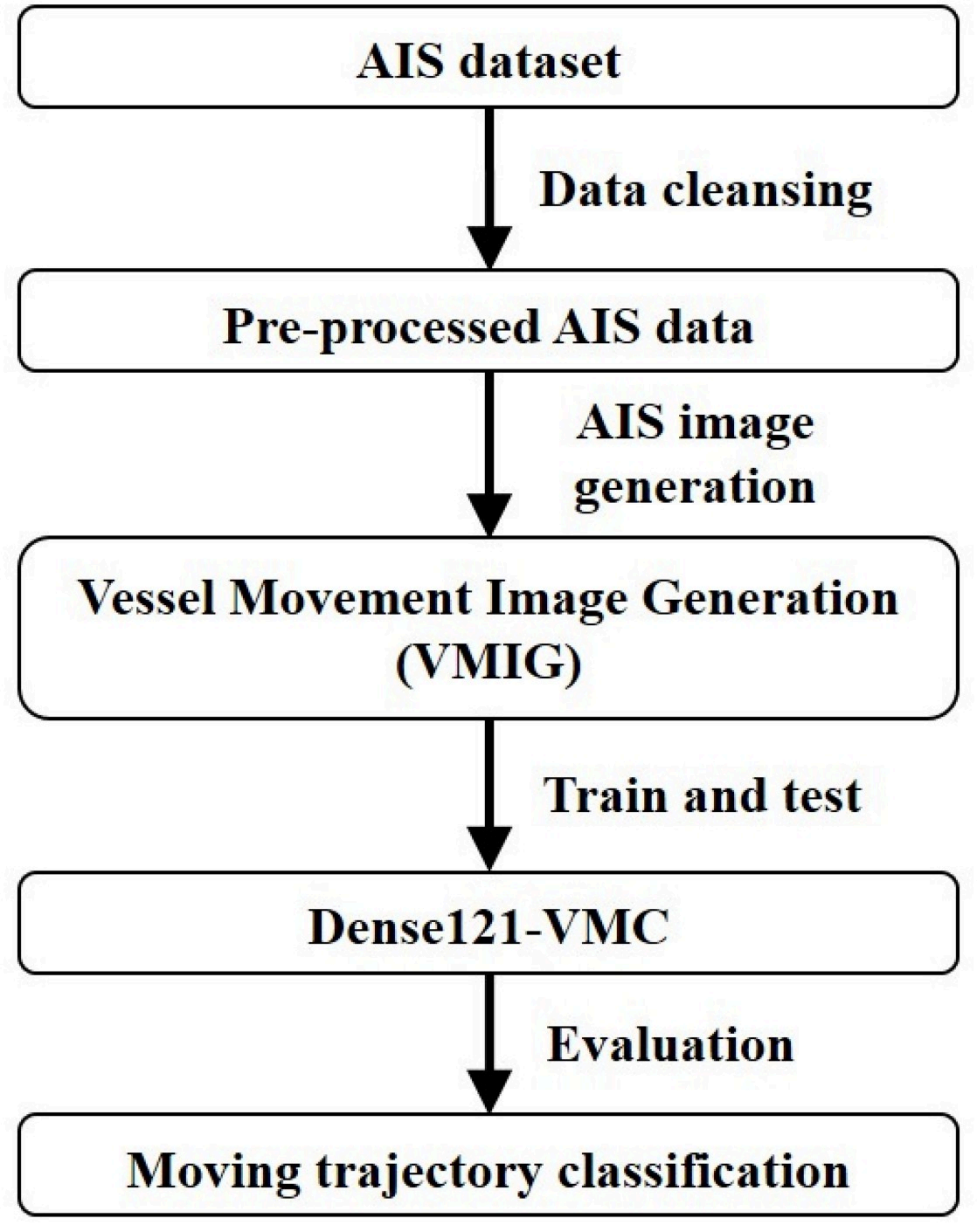
Workflow of vessel movement detection and classification.

In this section, we explain the details of the proposed method and overall workflow of AIS image generation and classification is well depicted in Fig 2. The extracted patterns from the previous step are converted into images by Pandas and the pixel of each image is 120 × 120.

### Vessel movement image generation (VMIG)

In this section, we introduce how the navigating and loitering patterns are exploited from AIS data and how these patterns are converted into AIS images for training.

#### Navigating pattern extraction

The AIS dataset is composed of the ship’s navigating information, and the important task is to discern subtle changes between turning and navigating patterns. In this work, [24] introduces an effective ship movement extraction algorithm. Building upon this algorithm, we extract the vessel’s navigating patterns. Let *T*_*r*_ = (*p*_1_, *p*_2_, …, *p*_*n*_) denotes a ship’s trajectory, where *n* represents the number of AIS points. Each AIS point is represented by a tuple *p*_*i*_ = (*MMSI*_*i*_, *t*_*i*_, *lat*_*i*_, *log*_*i*_, *sog*_*i*_, *cog*_*i*_), with *MMSI*_*i*_, Maritime Mobile Service Identity, for ship *i*, *t*_*i*_ denoting the time index, *lat*_*i*_ indicating latitude, *log*_*i*_ representing longitude, and *cog*_*i*_ expressing course of ground. The change rates in course between points *p*_*i*+1_ and *p*_*i*_ serve as the basis for determining the types of ship movements. The Eq 1 is employed to compute AIS point rates of changing course.

$$\begin{array}{c} △Cog=\frac{p_{i+1}\cdot cog_{i+1}-p_{i}\cdot cog_{i}}{t_{i+1}-t_{i}} \end{array}$$ (1)

**Fig 3:**
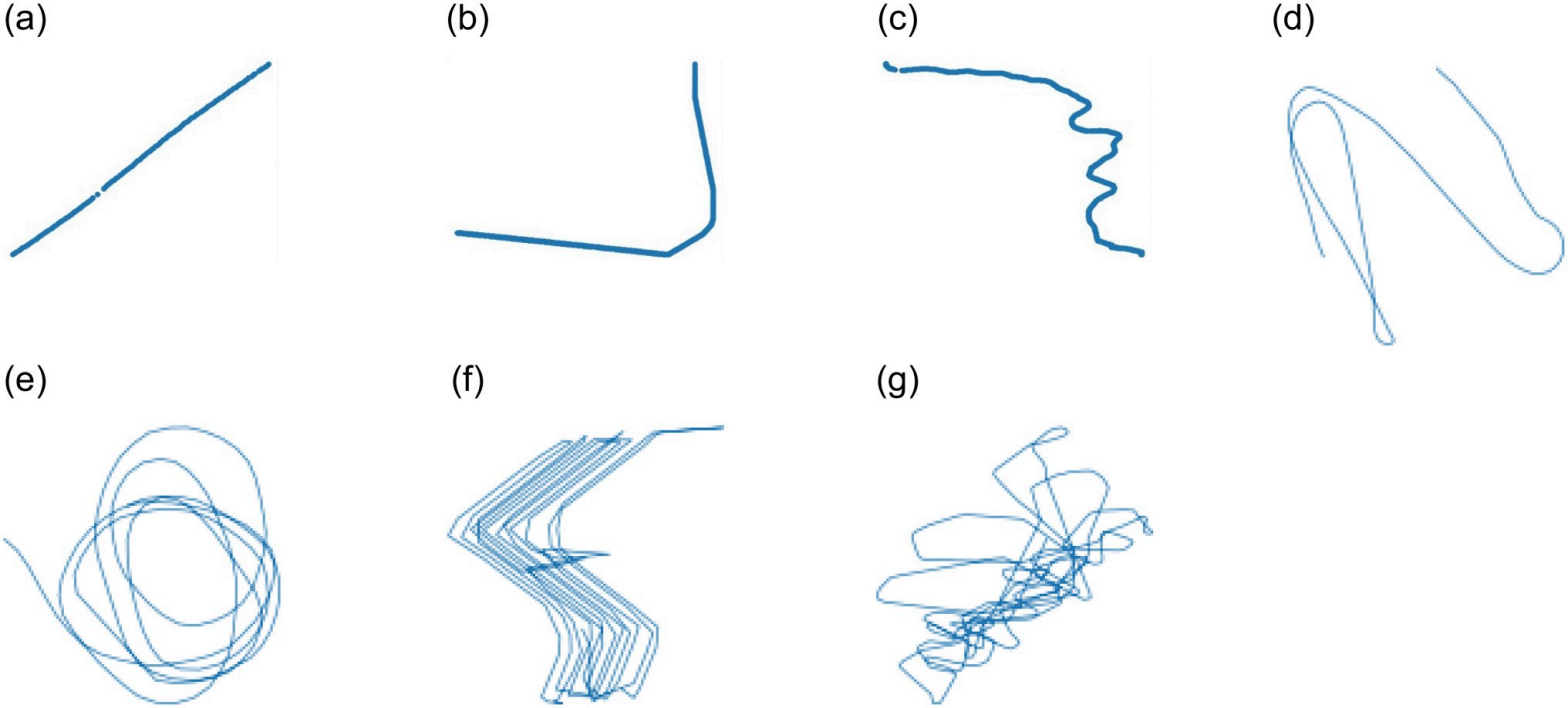
Seven typical movement patterns of vessel. **(a)** Straight-sailing. **(b)** Turning. **(c)** Navigating. **(d)** Loitering (disordered). **(e)** Loitering (lasso). **(f)** Loitering (regular). **(g)** Loitering (random).

Based on the calculated AIS point, if the △*Cog* is over the threshold, the current AIS point *p*_*i*_ is changing and it is a navigating state as shown in Fig 3(c).

#### Loitering pattern extraction

The term of trajectory redundancy is firstly proposed to quantitatively represent the distinctive trajectory features of ship loitering [8] and vessel loitering behaviors exhibit the two characteristics below. First, some ships can be categorized into the category of under-actuated objects, where spatial steering response speed and amplitude are constrained. The spatial range for ship loitering far exceeds that for pedestrian or vehicle loitering. Additionally, dynamic changes are influenced by the ship’s size. Secondly, factors such as wind, waves, currents, and other sailing conditions cause ship’s reciprocal motion characteristics in the small coverage. These factors can bring a certain level of interference in loitering recognition.

Hence, loitering behavior can be succinctly described as a limited scope of activities in the perimeter where a ship exists and as continuous alteration in navigation direction, bringing about a significant increasing rotation angle. To detect ship trajectories varying in spatial range and time duration, vessel loitering detection method based on multi-scale sliding windows can be leveraged efficiently. To effectively analyze this characteristic, this paper introduces the concept of trajectory redundancy to represent the density of trajectory and the frequency of course adjustments. Let *P* denote the boundary of the minimum size rectangle of the ship’s trajectory, serving as the reference value for the ship’s movement range. The length of the ship’s trajectory is represented as *D*. The trajectory redundancy, denoted as *θ*, is calculated using the following Eq 2.

$$\begin{array}{c} \theta =\frac{D}{P} \end{array}$$ (2)

The bigger *θ* is, the higher loitering behavior possibility. If *θ* ≥ 1, all trajectory points are considered as a part of loitering trajectory patterns. Subsequently, the detected set of loitering trajectories are categorized into four different types: disordered, lasso, regular, and random type, as shown in Fig 3(d)–3(g) respectively. On the other hand, if *α* ≤ *θ* < 1, where *α* is a parameter for straight-sailing pattern, every trajectory point is considered as general turning pattern as shown in Fig 3(b), and if *α* ≤ *θ*, then its points are classified as straight-sailing behavior as depicted in Fig 3(a).

### DCNN-based vessel movement classification

For vessel movement classification, we employ three types of pretrained DCNN models: ResNet, DenseNet, and Vision Transformer (ViT). Among the three types, ResNet and DenseNet are two well-known DCNN models in image classification tasks and ViT is based on Transformer model, which has achieved great success in the Natural Language Processing (NLP) area. Since DenseNet-121 model shows the most efficient performance among all models, we use this model for our framework: DenseNet-121 Vessel Movement Classification (Dense121-VMC). Due to the different sizes between trajectory images and input layer of models, we modify the size of the input layer. Likewise, because these models are pretrained for ImageNet datasets containing 1,000 classes, we change the dimension of the output layer.

## Experiments

In this section, we explain datasets and experimental settings used in this paper. Additionally, comprehensive experiments are conducted to prove the effectiveness of our method.

### Dataset

The AIS data employed in our experiment is available on the U.S. National Oceanic and Atmospheric Administration (NOAA) and the U.S. Bureau of Ocean Energy Management (BOEM) cooperative website. (https://marinecadastre.gov). Additionally, we use the Strait of Juan de Fuca area with latitude from 45°N to 50°N and longitude from 126°W to 122°W respectively. The AIS data in 2017 is leveraged and preprocessed for vessel movement classification task.

### Experimental settings

In order to train our model, we set image datasets for training (70%) and validation (30%). In addition, the number of total images used in the experiments is listed in Table 1. We set several hyper-parameter for our experiments, such as the number of epochs are 500, the Learning Rate is 1e-4, the probability of dropout *p*_*d*_ is 0.2, batch size is 32, and the Adaptive Moment (Adam) function is used for model optimization in this paper. Dense121-VMC is coded with Python, and we build the model based on PyTorch. We train our model on GPU, NVIDIA GeForce GTX 2080Ti.

**Table 1:** The number of images for vessel movement types in the training and validation stage.

Vessel movement types							
Normal Sailing				Loitering			
	Straight	Turning	Navigating	Disordered	Lasso	Regular	Random
Train	1,262	1,184	6,042	677	655	786	817
Test	541	508	2,590	290	281	336	350
Total	1,803	1,692	8,632	967	936	1,122	1,167

### Baselines

To demonstrate the effectiveness of Dense121-VMC in identifying trajectory patterns, we analyze our method with the following previous CNN-based approaches and DCNN-based methods:

- CNN-LSC [8]: It comprises 4 convolutional layers with several sizes of batch such as 16, 32, and 64.
- CNN-SMMC [24]: consists of 8 convolutional layers and one fully connected layer with batch normalization and pooling layers.
- ResNet-18: It includes a convolutional layer utilizing a 7 × 7 filter, followed by 16 convolutional layers employing a 3 × 3 filter with a fully connected layer. For each pair of 3 × 3 filters, a shortcut connection is introduced, forming a residual function.
- ResNet-50: It is composed of five sets, each set consists of 49 convolutional layers, along with a single fully-connected layer. The convolutional kernel encompasses three dimensions—1 × 1, 3 × 3, and 5 × 5—utilized for extracting features from the image at various resolution. Additionally, ReLU activation function follows all the convolutional layers.
- ResNet-101: In total, there are 104 convolutional layers. Specifically, it comprises a total of 33 layer blocks, and 29 of these blocks incorporate residual connections by directly utilizing the output from the preceding block. These residuals serve as the primary operand in the summation operation employed at the output of each block, forming the input for the subsequent blocks. The remaining 4 blocks take the output from the preceding block and apply it in a convolutional layer with a filter size of 1 × 1 and a stride of 1, followed by a batch normalization layer.
- ResNet-152: It comprises five sets, each containing 151 convolutional layers with a single fully-connected layer. The convolutional kernel encompasses three dimensions—1 × 1, 3 × 3, and 7 × 7—employed for extracting features from the image at various resolutions. Following the convolution layers, the activation functions used exclusively consist of the ReLU function.
- DenseNet-121: 1 7 × 7 convolutional layer, 58 3 × 3 convolutional layers, 61 1 × 1 convolutional layers, 4 average pooling layers, 1 fully connected layer.
- DenseNet-201: 1 7 × 7 convolutional layer, 98 3 × 3 convolutional layers, 101 1 × 1 convolutional layers, 4 average pooling layers, 1 fully connected layer.
- ViT-16: The Vision Transformer, known as ViT, is an image classification model utilizing a Transformer-like structure over 16 image patches. The image is divided into 16 patches, each undergoing linear embedding, with additional position embeddings. The sequence of vectors is then embedded in a conventional Transformer encoder. To facilitate classification, the standard technique involves incorporating an additional learnable “classification token” into the sequence.
- ViT-32: The Vision Transformer, also known as ViT, is an image classification model that utilizes a Transformer-similar structure over 32 image patches. The image is divided into 32 patches.

### Classification results

Table 2 shows the results between the proposed method and baselines on seven ship moving behaviors. DenseNet-121 records the highest accuracy on average and DenseNet-201 places the second highest average accuracy. We observe that DenseNet models more efficiently perform for image classification than ResNet models when these models go deeper. Previous CNN-based approaches, CNN-LSC and CNN-SMMC, show inferior performance to the deep CNN methods. It demonstrates that deep CNN models can extract features from input images more effectively than shallow models. Vision Transformer models, ViT-16 and ViT-32, report relatively poor performance. The possible reason is that its patches of images become similar as the number of patches increases since trajectory is expressed as a line in images and smaller lines look alike either straight or curve intuitively. Additionally, since ViT models basically require a lot of training datasets to properly train the model in classifying images, the small amount of training datasets could be an issue for vessel trajectory classification.

**Table 2:** Results of seven movement patterns (highest and second highest values are bold-faced and underlined, respectively).

Methods	Average Accuracy
CNN-LSC	92.64
CNN-SMMC	92.48
ResNet-18	94.3
ResNet-50	93.72
ResNet-101	94.34
ResNet-152	93.23
DenseNet-121	**95.79**
DenseNet-201	95.07
ViT-16	87.27
ViT-32	77.36

## Ablation study

In this section, we perform extended experiments and compare our approach with other methods to prove its superiority in identifying vessel trajectories.

### Comparison with CNN-based methods

In this section, we firstly compare Dense121-VMC model with state-of-the-art CNN-based methods, CNN-SMMC and CNN-LSC, on sailing and loitering moving patterns respectively.

#### Sailing movement patterns

**Fig 4:**
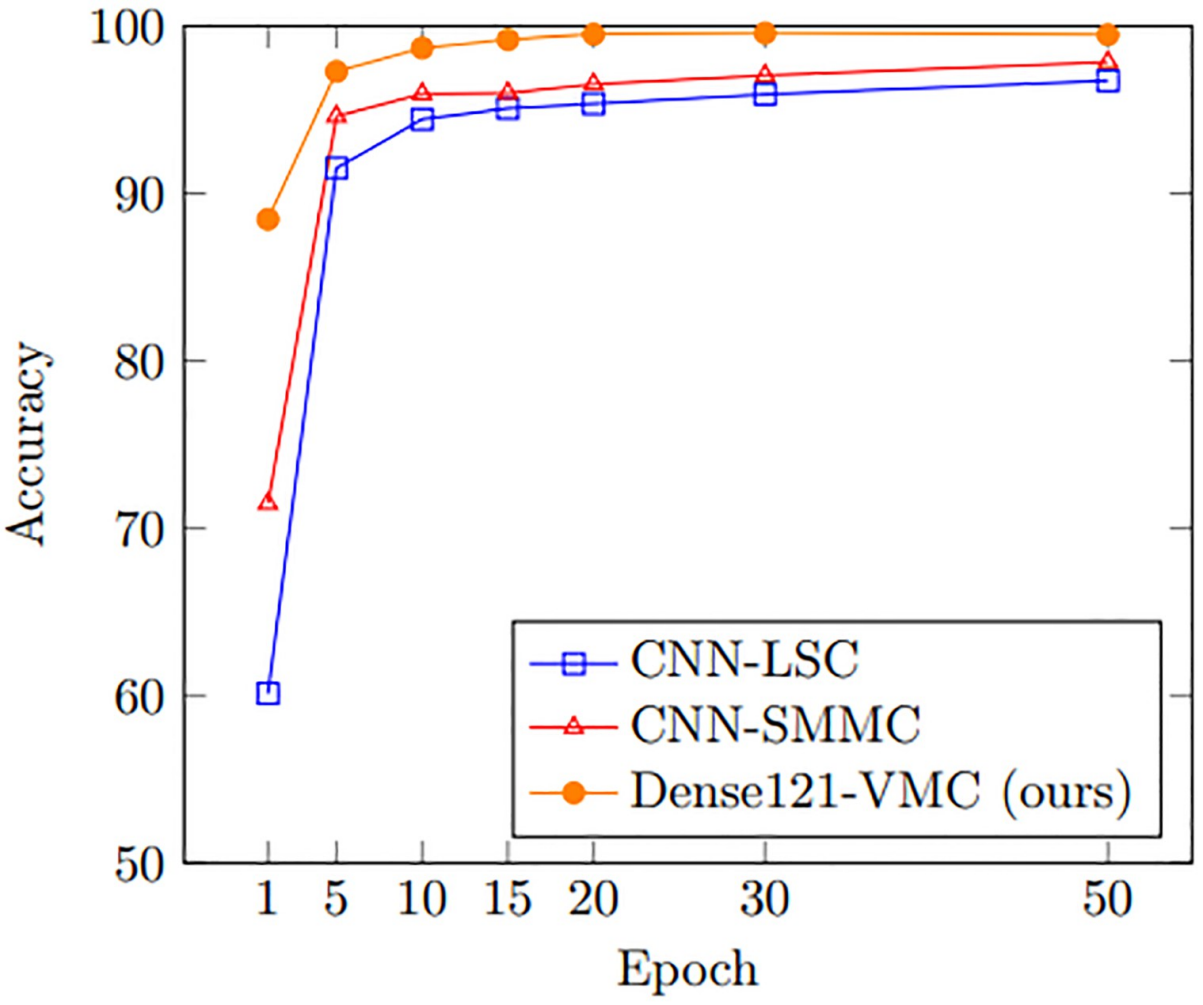
Performance comparison on three sailing patterns between previous CNN-based methods.

We analyze these CNN-based methods on three sailing patterns (Straight, Turning, and Navigating) to explore the most effective method among the previous methods. As shown in Table 3, proposed Dense121-VMC records 1.35% and 0.55% higher accuracy than CNN-SMMC and CNN-LSC respectively. This result indicates that the deeply stacked CNN layers are capable of identifying subtle differences of each trajectory image more efficiently than the previous shallow CNN models. In terms of training efficiency, our Dense121-VMC can be trained more efficiently as shown in Fig 4. In detail, Dense121-VMC shows its novel performance on every training epoch and converges its accuracy sooner.

**Table 3:** Accuracy statistics of three sailing pattern classifications.

Methods			
	CNN-SMMC	CNN-LSC	Dense121-VMC
Ave. Accuracy	96.09	96.89	**97.44**

#### Loitering movement patterns

**Fig 5:**
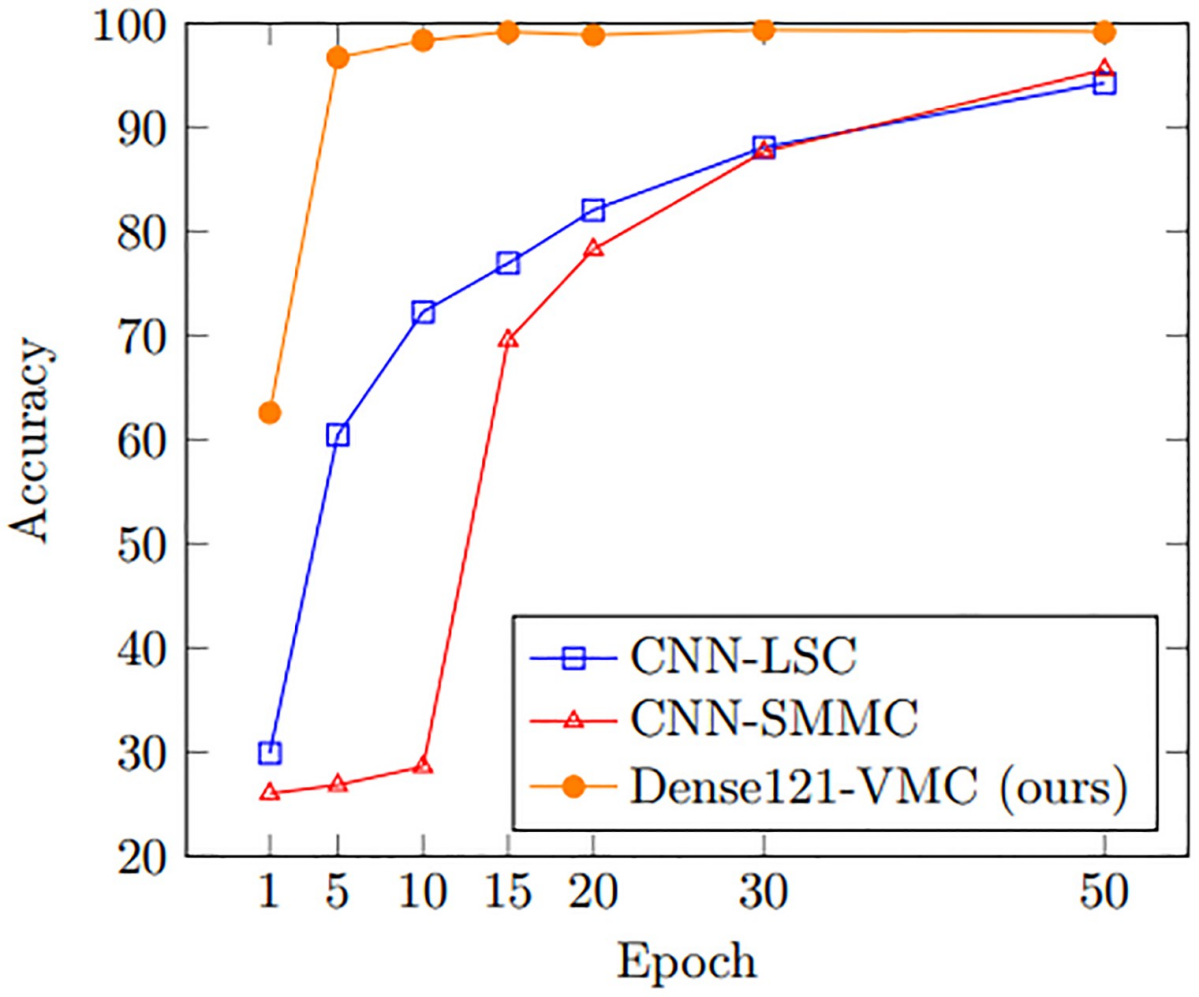
Performance comparison on four loitering patterns between previous CNN-based methods.

Firstly, we analyze the state-of-the-art CNN-based methods on four loitering behaviors (Disordered, Lasso, Regular, and Random) to explore the most efficient method among the methods. As listed in Table 4, our Dense121-VMC method shows 8.42% and 8.98% higher accuracy on average than CNN-SMMC and CNN-LSC respectively. When comparing with sailing pattern performance on previous section, we explore that performance of CNN-SMMC and CNN-LSC drops 10.48% and 11.84% respectively due to the class change from three to four classes. However, performance of our Dense121-VMC decreases only 3.41% and shows its robustness on the class change. Additionally, the performance of our method converges much faster than other approaches as shown in Fig 5. Our Dense121-VMC model shows its excellence in identifying loitering behaviors in comparison of state-of-the-art approaches.

**Table 4:** Accuracy statistics of four loitering pattern classifications.

Methods			
	CNN-SMMC	CNN-LSC	Dense121-VMC
Ave. Accuracy	85.61	85.05	**94.03**

### Comparison with DCNN models

In this section, we analyze the performance of the eight different DCNN models (ResNet, DenseNet, and ViT) on sailing and loitering movement patterns respectively.

#### Sailing movement behaviors

**Fig 6:**
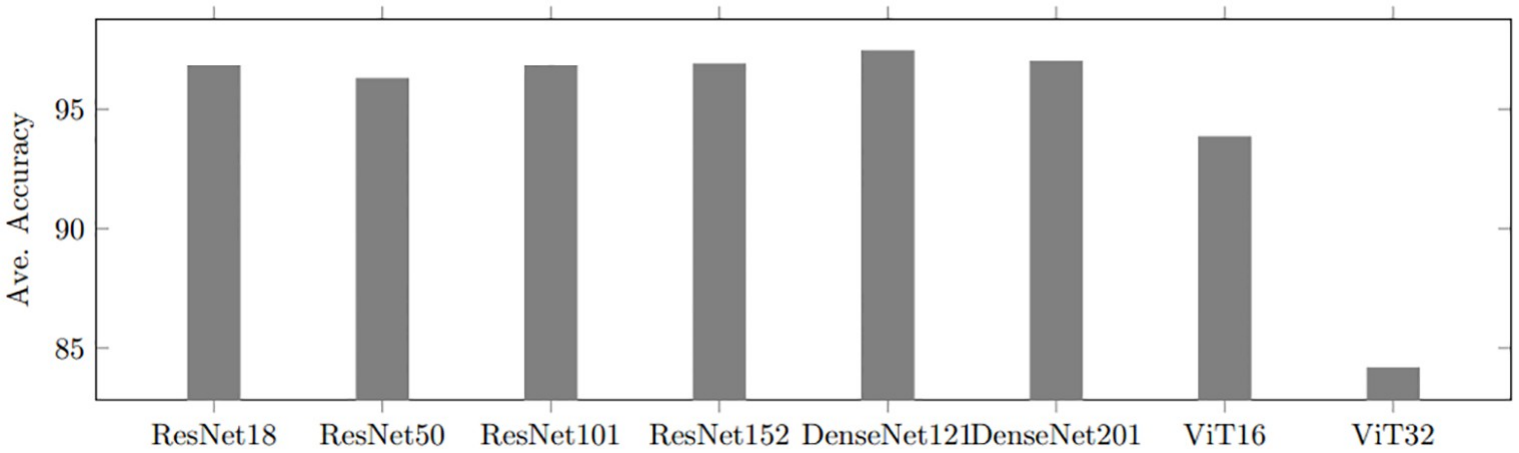
Performance comparison on three sailing patterns between DCNN-based methods.

To explore the best performance in classifying sailing patterns among the eight deep CNN models, we perform experiments on three sailing patterns (straight, turning, and navigating). DenseNet-121 model shows the highest accuracy for sailing pattern classification as depicted in Fig 6. The possible reason is that the connectivity pattern between CNN layers in DenseNet alleviates the gradient vanishing problem more effectively than ResNet. Meanwhile, ViT-32 records the least accuracy as the size of each patch becomes smaller and similar. The probable reason is that although ViT generally requires plenty amount of datasets in training, we train ViT models without enough amount of images. In addition, ViT splits images into patches. Then, as an image are divided into smaller patches, trajectory lines on each patch will become similar and similar. This is the reason that the performance of ViT-32 significantly drops in comparison with ViT-16. As a result, two DenseNet models, DenseNet-121 and -201, perform most effectively for three sailing movement identification among the eight deep CNN models.

#### Loitering movement behaviors

**Fig 7:**
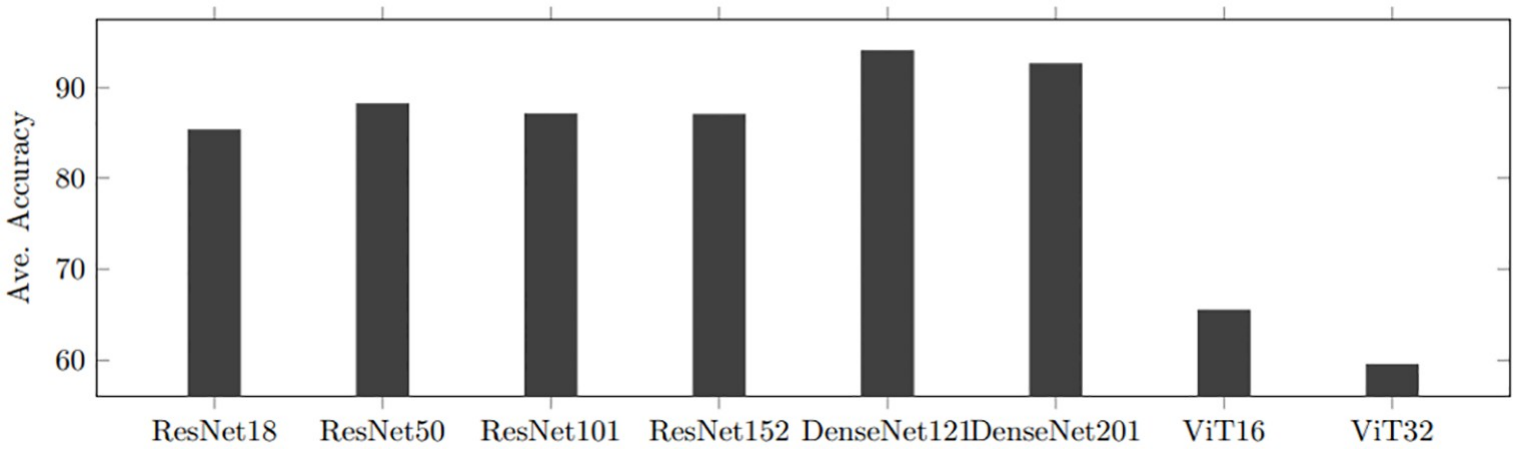
Performance comparison on four loitering patterns between DCNN-based methods.

To explore the best performance in classifying loitering patterns among the eight deep CNN models, we conduct experiments on four loitering types (disordered, lasso, regular, and random). As shown in Fig 7, DenseNet-121 reports the best accuracy. In addition, DenseNet models overall show the novel performance and ViT models report the lowest accuracy among ResNet, DenseNet, and ViT models. Particularly, the accuracy of ViT-16 seriously decreases as one more class is added. In general, ViT models exhibit remarkable performance on intricate images (e.g., ImageNet or CIFAR images). Since the trajectory images consist of simple lines, performance fluctuates as the number of patches changes due to the aforementioned reason. Consequently, DenseNet models work most efficiently among the eight deep CNN models for loitering movement classification.

### Comparison of RNN-based methods

**Fig 8:**
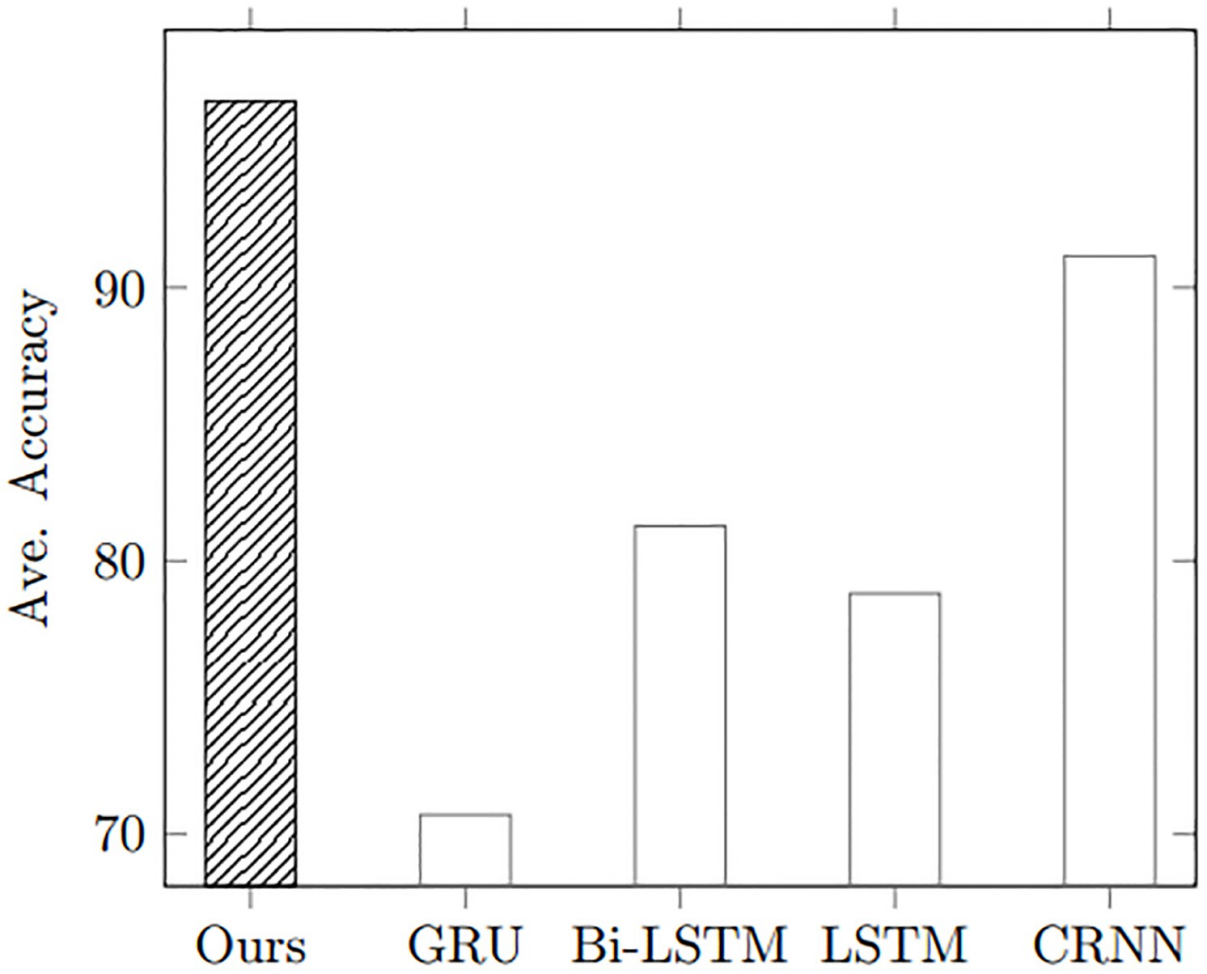
Classification results between our method and RNN methods.

All of temporal characteristics in time-series AIS data may not be disappeared when the AIS data is converted into an image, since vessel trajectories in the images originally stem from sequence of AIS data [30]. For example, if an image is converted from 1 hour AIS data, then the image shows patterns of ship movements for 1 hour. Our approach can effectively catch this temporal patterns without recurrent neural networks (RNNs). To demonstrate how the proposed method can efficiently handle the temporal information in images, we compare our approach with the existing recurrent-based methods. In this experiment, we choose 4 recurrent-based approaches [31–33]. As shown in Fig 8, our method proves its superiority in classification, while effectively handling temporal information in AIS images. In addition, CNN-RNN (CRNN) method shows second highest accuracy. This result can explain that our method can effectively handle the temporal patterns.

## Conclusion and future research

In this paper, we propose a new Dense121-VMC framework using pretrained DCNN models for vessel trajectory detection and classification. The sailing and loitering patterns are extracted from AIS points on the basis of angle distributions and ratio of course change. Then, these trajectory patterns are converted into 120 × 120 sized images. Dense121-VMC framework demonstrates its superiority to previous CNN-based approaches for ship trajectory classification. Our model is a pretrained model and efficiently classifies moving behaviors of vessels even without complex fine-tuning stages. For future research, we consider new directions as follows: (1) extracting more patterns of vessels, not only moving patterns but also mooring and anchoring behaviors, (2) fine-tuning of the pretrained DCNN models to alleviate its biases for performance enhancement, and (3) leveraging data augmentation methods, e.g., Generative Adversarial Networks (GANs), to improve the quality of the training and testing datasets.

## Supporting information

S1 File Our source code and datasets used in this research can be publicly available at online repository: https://github.com/missinghwan/AIS-classification. (TXT)
